# A Novel Splice Variant in the N-propeptide of *COL5A1* Causes an EDS Phenotype with Severe Kyphoscoliosis and Eye Involvement

**DOI:** 10.1371/journal.pone.0020121

**Published:** 2011-05-17

**Authors:** Sofie Symoens, Fransiska Malfait, Philip Vlummens, Trinh Hermanns-Lê, Delfien Syx, Anne De Paepe

**Affiliations:** 1 Center for Medical Genetics, University Hospital Ghent, Ghent, Belgium; 2 Department of Dermatopathology, University Hospital of Sart-Tilman, Liège, Belgium; Instituto de Ciencia de Materiales de Madrid - Instituto de Biomedicina de Valencia, Spain

## Abstract

**Background:**

The Ehlers-Danlos Syndrome (EDS) is a heritable connective tissue disorder characterized by hyperextensible skin, joint hypermobility and soft tissue fragility. The classic subtype of EDS is caused by mutations in one of the type V collagen genes (*COL5A1* and *COL5A2*). Most mutations affect the type V collagen helical domain and lead to a diminished or structurally abnormal type V collagen protein. Remarkably, only two mutations were reported to affect the extended, highly conserved N-propeptide domain, which plays an important role in the regulation of the heterotypic collagen fibril diameter. We identified a novel *COL5A1* N-propeptide mutation, resulting in an unusual but severe classic EDS phenotype and a remarkable splicing outcome.

**Methodology/Principal Findings:**

We identified a novel *COL5A1* N-propeptide acceptor-splice site mutation (IVS6-2A>G, NM_000093.3_c.925-2A>G) in a patient with cutaneous features of EDS, severe progressive scoliosis and eye involvement. Two mutant transcripts were identified, one with an exon 7 skip and one in which exon 7 and the upstream exon 6 are deleted. Both transcripts are expressed and secreted into the extracellular matrix, where they can participate in and perturb collagen fibrillogenesis, as illustrated by the presence of dermal collagen cauliflowers. Determination of the order of intron removal and computational analysis showed that simultaneous skipping of exons 6 and 7 is due to the combined effect of delayed splicing of intron 7, altered pre-mRNA secondary structure, low splice site strength and possibly disturbed binding of splicing factors.

**Conclusions/Significance:**

We report a novel *COL5A1* N-propeptide acceptor-splice site mutation in intron 6, which not only affects splicing of the adjacent exon 7, but also causes a splicing error of the upstream exon 6. Our findings add further insights into the *COL5A1* splicing order and show for the first time that a single *COL5A1* acceptor-splice site mutation can perturb splicing of the upstream exon.

## Introduction

**Figure 1:**
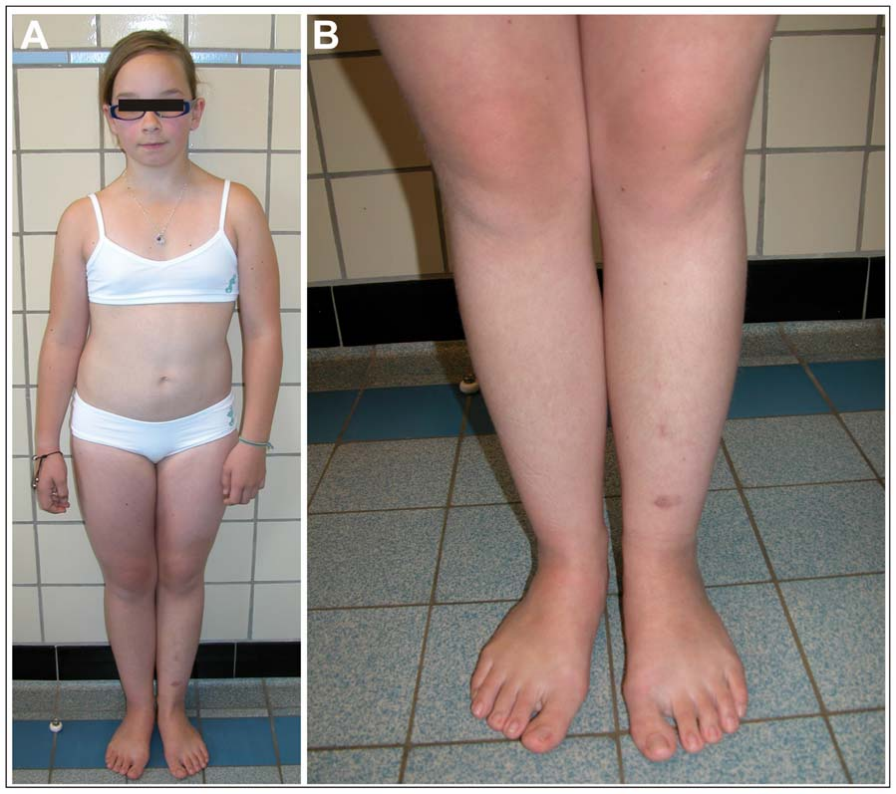
Clinical pictures of the patient. A–B. The patient at age of 12 yrs 4 months, presenting marked scoliosis, mild pectus excavatum and broad, flat feet. Scarring is only minimal but she presents hemosiderine deposits on the lower limbs.

Type V collagen, a quantitatively minor fibrillar collagen that is widely distributed among connective tissues, plays an important role in the organization of heterotypic type I/V collagen fibrils [1]. Its major isoform is the [α1(V)]_2_α2(V) heterotrimer, composed of two proα1-chains and one proα2-chain [1], [2]. The ability of type V collagen to regulate the collagen fibril diameter has been attributed to its amino(N)-propeptide [1]. The α1(V)-N-propeptide is a multidomain region, composed of an N-terminal TSPN-1 (thrombospondin-1 N-terminal domain like) domain, a variable (VAR) domain, a small interrupted collagenous domain (COL2) and a non-collagenous (NC2) domain (Figure 1A), whereas the α2(V)-N-propeptide consists of only one cysteine-rich (CR) domain. *In vivo*, type V procollagen processing by Bone Morphogenetic Protein-1 (BMP-1) releases the TSPN-1 domain from the proα1(V)-chain and removes the carboxyl(C)-propeptide from the proα2(V)-chain. Apparently, the α2(V)-N-propeptide is not processed by any protease, while the proα1(V)-C-propeptide is cleaved by furin. As such, the mature α1(V)-chain retains the VAR, COL2 and NC2 domains and the mature α2(V)-chain retains the CR domain at its N-terminus [3]. These domains are thought to regulate the diameter of heterotypic type I/V collagen fibrils [1].

Defects in the proα1(V)- or the proα2(V)-chain, encoded by *COL5A1* and *COL5A2* respectively, lead to classic Ehlers-Danlos Syndrome (EDS), formerly known as EDS type I, “gravis” (MIM# 130000) and EDS type II, “mitis” (MIM# 130010). This autosomal dominant disorder is characterized by hyperextensibility of the skin, delayed wound healing, atrophic scarring and generalized joint hypermobility. Typically, large composite collagen fibrils are observed in the dermis of classic EDS patients (collagen “cauliflowers”) [4]. The majority of *COL5A1* mutations introduce a premature stopcodon, resulting in nonsense-mediated mRNA decay and decreased type V (pro)collagen production [5], [6]. A minority of mutations causes the production of structurally abnormal type V collagen, the secretion of which may be impaired but not completely abolished [5], [6], [7]. We previously reported two other types of defects in type V collagen that prevent incorporation of the mutant α-chain into the [α1(V)]_2_α2(V) heterotrimer, respectively deletion of one or more cysteine residues in the α1(V)-C-propeptide and signal peptide mutations that impair secretion of the mutant α1(V)-chain [7], [8], [9]. So far only two mutations resulting in EDS have been described within the highly conserved α1(V)-N-propeptide (Figure 1A) [10], [11]. The first mutation, p.Gly530Ser, is located in the COL2 domain and is thought to be disease-causing when present in homozygous state, and disease-modifying when occurring in heterozygous state [10]. The second mutation, IVS4-2A>G, is an acceptor-splice site mutation and results in several in-frame splice transcripts [11]. In the two major splice forms, the BMP-1 cleavage site of the α1(V)-N-propeptide is deleted, which results in the retention of the TSPN-1 domain [11].

In the current study, we report a novel *COL5A1* acceptor-splice site mutation in intron 6 (IVS6-2A>G, NM_000093.3_c.925-2A>G) that affects splicing of exon 7 and the upstream exon 6 and causes an EDS phenotype, characterized by mild cutaneous involvement, eye involvement and severe progressive kyphoscoliosis.

## Results

### Clinical findings

**Figure 2:**
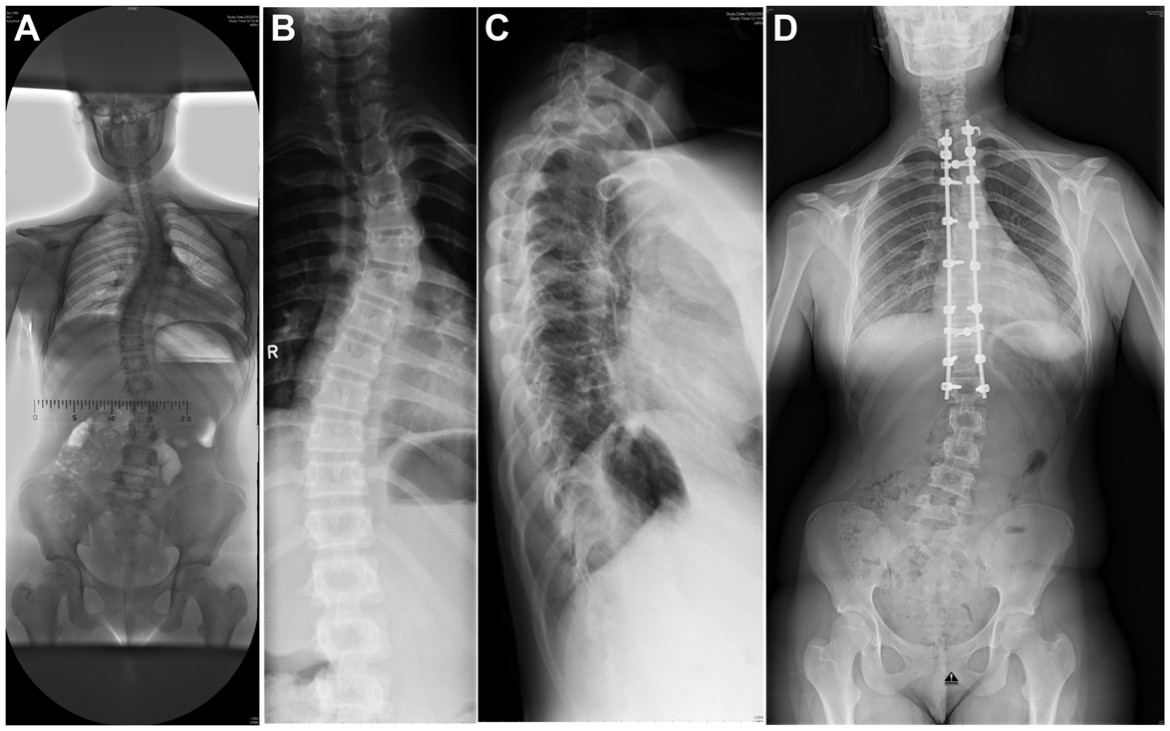
X-ray photographs of the thorax of the patient. A. Severe scoliosis. B. Close-up of the thoracic scoliosis. C. X-ray side view showing the kyphosis of the patient. D. X-ray of the thorax of the patient after corrective surgery for the progressive scoliosis.

The patient is the only child of healthy non-consanguineous parents (Figure 1A). She was born at 31-weeks of gestation after an uneventful pregnancy and premature rupture of the membranes. Her birth length was 46 cm, birth weight was 2050 g and head circumference was 38 cm at two months of age. She presented marked joint hyperlaxity. Neuromotor development was normal, although she gave a clumsy impression. At the age of 6 years she sustained a supracondylar humerus fracture after a fall. Her skin was fragile, with delayed wound healing and easy bruising. In addition to amblyopia and hypermetropia, she had converging strabism for which she was operated twice. She suffered from retinal detachment in the right eye. At age 9 years, she developed a severe, rapidly progressing kyphoscoliosis (Figure 2A–C).

Clinical examination at the age of 12 years and 4 months showed a girl with a length of 141.3 cm (P3), weight of 44 kg (P50) and head circumference of 51.6 cm (P50). Inspection of the facial features revealed prominent ears, light-blue sclerae, bilateral epicanthic folds, mild micrognathia and dental crowding (Figure 1A). She had very soft, velvety and hyperextensible skin, with multiple ecchymoses and hemosiderine deposits on the lower limbs (Figure 1B). Scarring was minimal with only one small atrophic scar on the left elbow. Small molluscoid pseudotumors were present on the side of her feet. She presented generalized joint hypermobility with a Beighton score of 7/9, marked kyphoscoliosis, mild pectus excavatum and broad, flat feet.

At age 13^1/2^ years her main problems included fatigue, muscle weakness with reduced physical capacity and rapidly progressive kyphoscoliosis, for which she had corrective surgery (Figure 2A–D). Clinical evaluation four months after surgery showed that the scoliosis correction remained stable. Echocardiography was normal.

### Biochemical and Ultrastructural Collagen Studies

**Figure 3:**
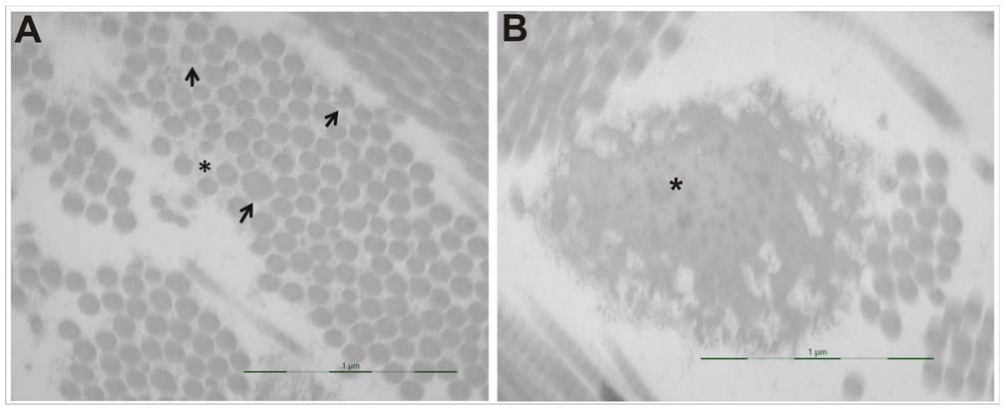
Ultrastructural electron microscopy studies. A. In addition to the high variability in diameter of the collagen fibrils, cauliflower-like collagen fibrils (arrows) and granulo-filamentous deposits (*) in the interfibrillar spaces are seen. B. Elastic fiber with increased osmiophilic elements in the elastin matrix (*).

SDS-PAGE analysis of procollagens and pepsin-digested collagens from the cell layer and medium fraction showed a normal electrophoretic pattern for types I and III (pro)collagen. Unfortunately, no definite conclusion could be drawn for type V collagen as the bands representing the α1(V)- and α2(V)-chains were too low in intensity (data not shown). This finding is in agreement with a previous report demonstrating that biochemical analysis is a poor diagnostic test for detecting type V collagen abnormalities [5]. Ultrastructural studies revealed collagen bundles with fibrils of variable diameter and irregular interfibrillar spaces enriched in granulo-filamentous deposits (Figure 3A). We observed numerous cauliflower-like collagen fibrils, a hallmark of disturbed collagen fibrillogenesis, typically found in the dermis of classic EDS patients (Figure 3A). While most elastic fibers presented normal ultrastructural features, in some osmiophilic elements were increased (Figure 3B). In addition, the dermal fibroblasts of the patient exhibited a dilated endoplasmic reticulum (data not shown).

### Molecular analysis

**Figure 4:**
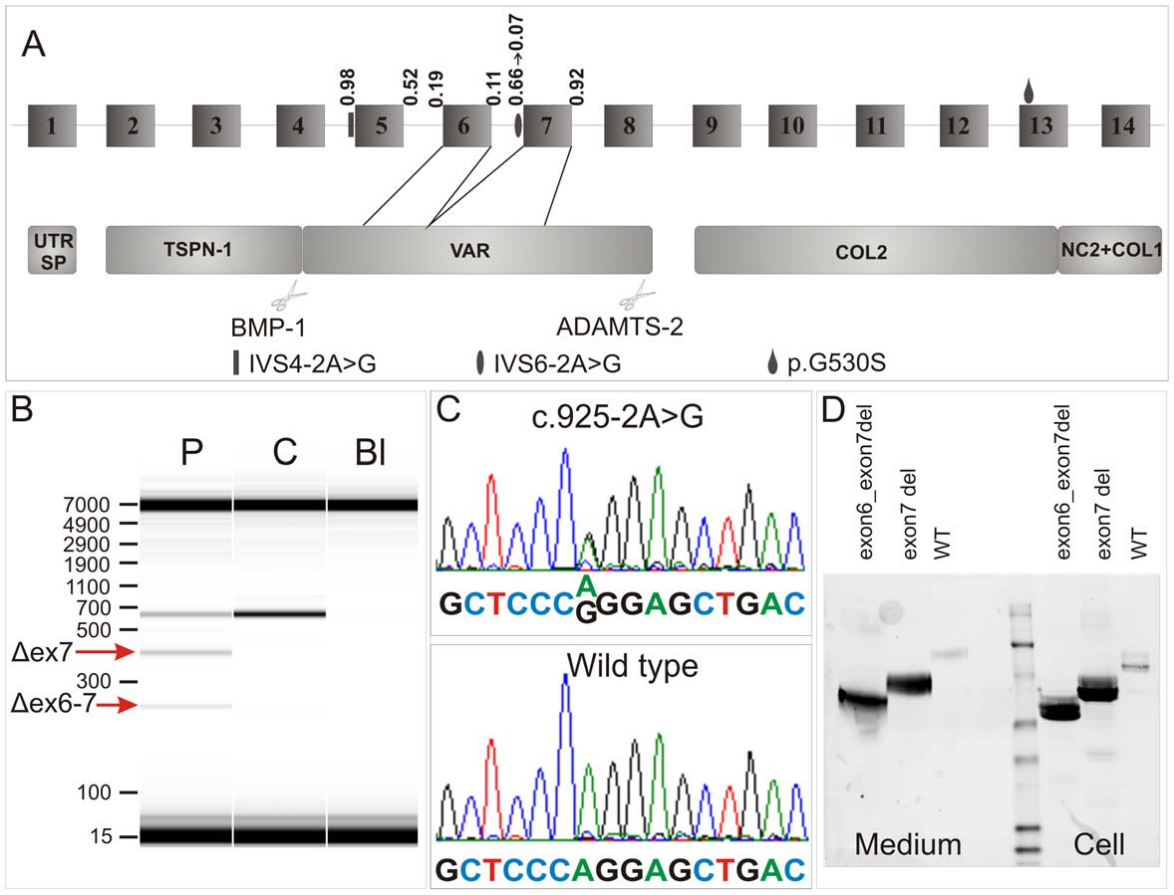
NM_000093.3_c.925-2A>G (IVS6-2A>G) mutation data. A. Exon and domain structure of the *COL5A1* N-propeptide encoding region. Known mutations and the NM_000093.3_c.925-2A>G (IVS6-2A>G) mutation are indicated. Deletion of exon 7 and deletion of exon 6 and 7 affect the VAR domain of the pro-α1(V)-N-propeptide. B. RT-PCR was performed using primers located in exon 4 (828F) and exon 8 (1427R). Three different products are present in the patient sample (P). The upper band corresponds to the wild type product, the middle band corresponds to the product in which exon 7 is deleted, and the lower band corresponds to the product in which both exon 6 and exon 7 are deleted. These bands were cloned and sequenced. In the control sample (C) only the wild type product is detected. C. Electropherogram of the NM_000093.3_c.925-2A>G (IVS6-2A>G) mutation. D. Western blot analysis of HEK293-T transfected with WT, exon7deletion-pNα1(V)-pCEP4 and exon6_exon7deletion-pNα1(V)-pCEP4 constructs respectively. All three constructs are translated (Cell) and efficiently secreted into the extracellular matrix (Medium).

In view of the rapidly progressing kyphoscoliosis and ocular symptoms, mutation screening of *PLOD1* was initially performed to exclude the kyphoscoliotic type of EDS (EDS, type VIA), but no *PLOD1* mutation was identified. Considering her fragile skin with delayed wound healing, further molecular studies were directed towards the type V collagen genes. A *COL5A1* null-allele test showed that both *COL5A1* alleles were expressed, thereby excluding the presence of a *COL5A1* null-allele. *COL5A1* mutation screening identified a heterozygous A-to-G transition in the acceptor-splice site of exon 7 (IVS6-2A>G, NM_000093.3_c.925-2A>G) (Figure 4A–C), which was absent in the unaffected mother. Unfortunately, gDNA of the patients' unaffected father was unavailable. Mutation screening of *COL5A2* was normal.

The NM_000093.3_c.925-2A>G substitution abolished the *COL5A1* exon 7 acceptor-splice site, as predicted by the Splice Site Prediction by Neural Network software. HSF analysis confirmed disruption of the acceptor-splice site and predicted that the potential branch point of intron 6 would be broken. In addition, the HSF software predicted that the NM_000093.3_c.925-2A>G mutation can interfere with binding of several SR proteins, such as SC35, SRp40 and SF2/ASF, to SREs, which may cause malpositioning of the spliceosome.

The ExonScan software was used to predict potential exons in the entire *COL5A1*-N-propeptide encoding region (spanning exons 1 to 14). Interestingly, while exons 2, 3, 4, 8, 9, 10, 11 and 13 were correctly predicted, exons 1, 5, 6, 7, 12 and 14 were not recognized. The fact that, despite their weak splice sites, these exons are recognized *in vivo* by the spliceosome suggests that other sequences or pre-mRNA secondary structures are involved in directing the spliceosome to these exons. Subsequently, the Mfold program was used to determine local pre-mRNA secondary structures surrounding the exon 7 acceptor-splice site. We evaluated an 80-mer containing the exon 7 acceptor-splice site and neighboring sequences. In the wild type allele, the exon 7 acceptor-splice site was present in a “loop” structure and thus easily accessible for the splicing machinery (Supplementary Figure S1). In contrast, in the presence of the NM_000093.3_c.925-2A>G mutation, the pre-mRNA secondary structures changed and the acceptor-splice site shifted towards a “stem” structure, making this site less accessible for the spliceosome (Supplementary Figure S1).

To investigate whether the NM_000093.3_c.925-2A>G mutation gave rise to different *COL5A1* mRNA transcripts, we cloned the mRNA fragment comprising exons 4 to 8 (Figure 4A–B). Two different mutant mRNA transcripts were detected, which were present in equal amounts: one transcript lacked exon 7, the second transcript missed exon 7 and the upstream exon 6 (Figure 4A–B). Deletion of exon 7 and of exons 6 and 7 removed 80 and 126 amino acids respectively from the VAR domain.

### Expression and secretion of wild type and mutant pNα1(V)

Next, we investigated whether both mutant transcripts were translated and secreted into the extracellular matrix. The wild type expression plasmid encoded the entire α1(V)-N-propeptide domain (pNα1(V)-pCEP4) [12], while the first mutant expression construct lacked exon 7 (exon7deletion-pNα1(V)-pCEP4) and the second lacked exons 6 and 7 (exon6_exon7deletion-pNα1(V)-pCEP4). Immunoblotting experiments on the cell and medium fraction of transfected HEK293-T cells demonstrated that, analogous to the wild type protein, both aberrant proteins were produced and efficiently secreted into the extracellular matrix (Figure 4D). As such, these mutant α1(V)-chains are thought to be incorporated into type V collagen heterotrimers and to disturb normal type I/V collagen fibrillogenesis, as illustrated by the presence of collagen cauliflowers observed in the patients' dermis.

### Determination of Intron Removal

**Figure 5:**
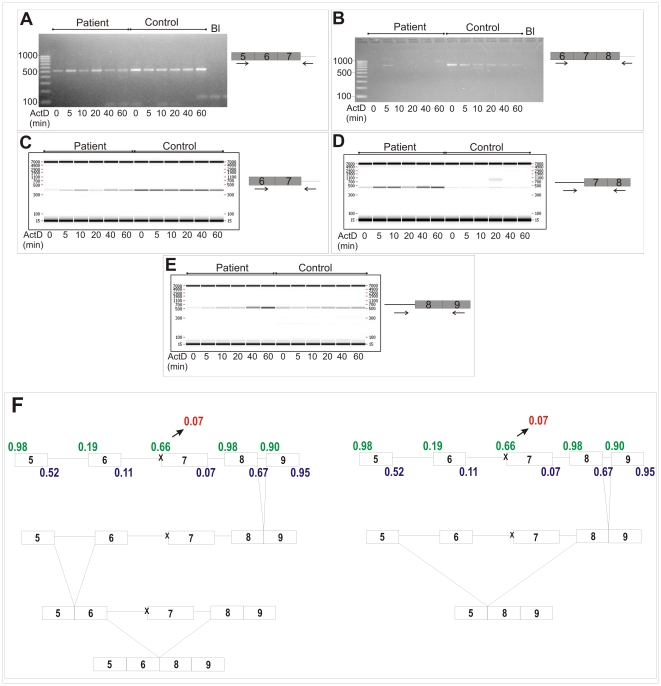
Determination of the order of intron removal in the region between exons 5 and 9 on partially spliced *COL5A1* pre-mRNA (cDNA). Nuclear RNA was isolated from cultured fibroblasts after incubation with Actinomycine D (ActD) for 0, 5, 10, 20, 40 and 60 min to stop transcription. A. For both the patient and the control sample a persistent PCR product was observed, implying that intron 7 is slowly removed. B. The exon6/intron 8 primer pair resulted in a gradually decreasing PCR band in the control sample. This band is not visible in the patient. We conclude that intron 8 is removed quickly in both the control and the patient sample; but intron 8 is removed more quickly in the patient sample when compared to the control sample. C. PCR with primers in exon 6 and intron 7 showed a persistent PCR band for both control and patient sample, suggesting that intron 6 is removed prior to intron 7. D. The intron6/exon8 primer pair resulted in a distinctly visible band for the patient sample, while for the control sample only a very weak PCR signal was obtained. This suggests that the NM_000093.3_c.925-2A>G mutation causes delayed splicing of intron 6. E. Intron 7/exon9 primers showed a weak persistent PCR band in both control and patient sample. Moreover, in the patient sample, an accumulation in time was seen. As such, it appears that the NM_000093.3_c.925-2A>G mutation also delays splicing of intron 7. F. Predicted pathways of intron removal in the mutant COL5A1 allele (x indicates the location of the NM_000093.3_c.925-2A>G mutation). Intron 8 is quickly removed. Subsequently, two pathways are possible. In the first pathway, intron 5 is removed, leading to a fused exon5/exon 6. The next step is skipping of exon 7 (left side of the panel). In the second pathway (right side of the panel), the weak acceptor-splice site of exon 6 is not used by the spliceosome. Instead, the combined use of the donor-splice site of exon 5 with the acceptor-splice site of exon 8 leads to skipping of both exons 6 and 7. Green, acceptor-splice site; blue, donor-splice site; red, mutated exon 7 acceptor-splice site.

To determine the order of intron removal in the region between exons 5 and 9, paired intron/exon primers were used on nuclear pre-mRNA splicing intermediates. When an exon 5/intron 7 primer pair was used (Figure 5A), a PCR product remained present for 60 min in the patient and control cells. This PCR fragment corresponded to the mRNA product in which both intron 5 and 6 had been removed, but in which intron 7 was still present. We concluded that intron 7 is removed more slowly from the mRNA than intron 5 and intron 6.

When using an exon 6/intron 8 primer pair (Figure 5B), a PCR band, in which intron 6 and 7 are removed, was visible in the control sample as a gradient line which decreases in time. However, this band was not observed in the patient sample. We concluded that, while in the control sample intron 8 is removed from the pre-mRNA in a time-dependent manner, in the patient sample removal of intron 8 is accelerated and removal of intron 6 and/or 7 is delayed. Similar results were obtained with the primer pair exon 5/intron 8 (data not shown).

Additional studies with primers in exon 6 and intron 7 (Figure 5C) showed a persistent PCR signal in both samples, consistent with the slow removal of intron 7. This suggests that intron 6 is removed prior to intron 7.

Primers located in intron 6 and exon 8 (Figure 5D) yielded a PCR product in which intron 7 is removed. In the control sample only a low intensity PCR signal was visible. But, in the patient sample a persistent PCR signal with higher intensity was detected, indicating that pre-mRNA splicing intermediates, still containing intron 6, accumulate. As such, the splice acceptor-mutation causes delayed splicing of intron 6. In addition, primers located in intron 7 and exon 9 (Figure 5E) demonstrated the slow removal of intron 7 in both the control and the patient sample, again confirming that intron 7 is a slowly removed intron. A more intense PCR signal was observed after 40 and 60 minutes in the patient sample when compared to control, indicating that in the presence of the NM_000093.3_c.925-2A>G mutation splicing of intron 7 is even more delayed (Figure 5E). This implies that the NM_000093.3_c.925-2A>G mutation might not only disturbs splicing of the affected intron 6, but also further delays splicing of the already slowly spliced-out intron 7.

These data are consistent with a model implicating at least two pathways of intron removal between exons 5 and 9 in the presence of the NM_000093.3_c.925-2A>G mutation (Figure 5F). Our results indicate that the first splicing event is removal of intron 8. Subsequently, at least two orders of splicing are possible. In the first pathway (left side of figure 5F), the acceptor-splice site of exon 6, although predicted to be weak (strength 0.19), is recognized leading to removal of intron 5, thereby generating an mRNA splicing intermediate with a fused exon 5/exon 6 and in which exon 7 is flanked by intron 6 and 7. Due to the presence of the NM_000093.3_c.925-2A>G mutation, the acceptor-splice site of exon 7 is abolished, resulting in skipping of exon 7.

In the second pathway (right side of figure 5F), the spliceosome does not recognize the acceptor-splice site of exon 6, possibly due to changes in the secondary structure of the pre-mRNA induced by the mutation. Neither does it recognize the mutated acceptor-splice site of exon 7. Therefore the spliceosome uses the donor-splice site of exon 5 and the acceptor-splice site of exon 8. This causes the generation of the two-exon-skip transcript, lacking both exon 6 and 7.

The complexity of intron removal in the N-propeptide encoding region of the *COL5A1* gene is further underscored by the study of Takahara et al., 2002 [11], which showed the presence of several pathways of intron removal, resulting in a complex splicing outcome of the IVS4-2A>G mutation. Furthermore, we and others [11] used only part of the sequence of the entire *COL5A1* gene, thereby possibly excluding other regions present in *COL5A1* which may contribute to the observed splicing pattern. Combining the findings presented here with previous results suggest that other pathways of intron removal may exist. This may be supported by the fact that we were unable to detect the higher molecular weight PCR bands, corresponding to nuclear mRNA in which all introns remain present.

## Discussion

Most reported type V collagen defects cause the generation of a non-functional *COL5A1* allele, while only a minority lead to structurally abnormal type V collagen (i.e. glycine substitutions and splicing errors in the collagen triple helix, C-propeptide mutations). Interestingly, most of the currently identified type V collagen defects seem to act in a similar way, causing “functional” haploinsufficiency of type V collagen [7]. We present here a novel *COL5A1* acceptor-splice site mutation which is located in the N-propeptide domain, a region playing a significant role in the regulation of collagen fibril diameter [1]. We show that this mutation results in the generation of two mutant transcripts which are each predicted to generate a truncated protein. Even though we did not detect these truncated type V collagen proteins with routine biochemical SDS-PAGE analysis, the production and secretion of both truncated proteins in our recombinant expression system and the presence of collagen cauliflowers in the patients' dermis (Figure 3A), a hallmark of disturbed collagen fibrillogenesis, suggest that these truncated proteins are indeed produced and secreted into the extracellular matrix where they interfere with collagen fibrillogenesis. Although the N-propeptide domain is very highly conserved, this mutation is only the third mutation reported in this domain [10], [11]. Furthermore, our detailed study of the splicing outcome of this acceptor-splice site mutation adds to the growing number of studies investigating complex splicing outcomes and determining additional factors needed for correct pre-mRNA splicing. In contrast to other type V collagen mutations, the associated phenotype is not typical for classic EDS but differs from it in the mild cutaneous involvement, rapidly progressive, severe kyphoscoliosis (Figure 1A–D) and ocular complications. In view of the latter, molecular analysis of the *PLOD1* gene was performed, which excluded its contribution to the phenotype.

As demonstrated by numerous studies, several factors determine the fidelity of pre-mRNA splicing; e.g. four intronic core splicing signals (donor-splice site, acceptor-splice site, polypyrimidine tract and branch point), splicing regulatory elements (SREs), trans-acting factors and additional characteristics such as splice site strength, intron/exon architecture, pre-mRNA secondary structure, extracellular signaling and the transcription process itself (e.g. the C-terminal domain of RNA polymerase II is required for efficient pre-mRNA processing) [13], [14], [15], [16], [17]. While the majority of the hitherto characterized pre-mRNA splicing defects, involved in heritable disorders, reside in the donor- and acceptor-splice sites, recent studies have documented several mutations with a very complex splicing outcome [11], [18], [19], [20], [21], [22]. Our study expands this latter group of mutations. The novel *COL5A1* acceptor-splice site mutation, NM_000093.3_c.925-2A>G (IVS6-2A>G) generates two mutant transcripts, one with an exon 7 skip and one with a two-exon-skip of exon 7 and the upstream exon 6 (Figure 4A–B). Several computational calculations predicted that the IVS6-2A>G mutation interferes with the normal splicing process and leads to a profound change in pre-mRNA secondary structure surrounding the exon 7 acceptor-splice site (Supplementary Figure S1). Interestingly, a previously reported *COL5A1*-acceptor-splice site mutation (IVS4-2A>G) does not only affect the splicing of exon 5, but also of the downstream exon 6 [11], and also generates two minor transcripts by using a cryptic splice site in exon 5. The splicing outcome of the IVS4-2A>G mutation is of particular interest in relation to our results. More specifically, none of these three “affected” exons (exon 5–6–7) were predicted as possible exons by the ExonScan software, an algorithm based on splice site strength and the presence of splicing regulatory elements. This implies that additional factors are needed for the correct guidance of the spliceosome to these three exons, a hypothesis that is further supported by the findings published by Takahara et al., 2002 [11]. However, conversely, the previous study [11] suggested that, when splice site mutations lead to the generation of a two-exon-skip, the exons chosen to be skipped would differ depending on whether the mutation is at an acceptor-splice site or a donor-splice site. This would mean that an acceptor-splice site mutation leads to removal of the two downstream exons, while a donor-splice site mutation would allow skipping of the two exons upstream from the mutation site. However, as the mutation described here affects an acceptor-splice site and leads to skipping of the upstream exon in the two-exon-skip outcome, these findings illustrate that the exons involved in the two-exon-skip splicing outcome do not solely depend on whether the mutation affects an acceptor- or donor-splice site. Interestingly, although the IVS6-2A>G mutation, presented here, and the IVS4-2A>G mutation [11] are located very closely to each other, it is unclear why these patients differ in clinical phenotype. While our patient has rather atypical findings of classic EDS, the patient described by Takahara et al., 2002 [11] presented with typical signs of classic EDS (type I, gravis type) such as very soft, hyperextensible skin, many scars on his forehead and shins, and generalized small and large joint hypermobility.

We also analyzed the mechanism whereby the present mutation caused the generation of two different mutant transcripts in more detail by determining the order of intron removal. Interestingly, our studies showed that the mutation did not only abolish splicing of intron 6, but also delayed the removal of intron 7. When comparing the patient with the control, an intranuclear accumulation in time was observed for the pre-mRNA fragment in which intron 7 was retained. While all other introns were removed appropriately, failure to excise intron 6 and delay in splicing of intron 7 can explain the occurrence of the exon 7 skip (Figure 5F left panel). The occurrence of the two-exon-skip cannot solely be explained by these factors, which suggests that beside the order of intron removal, also changes in the pre-mRNA secondary structure hamper the spliceosome in recognizing the already weak acceptor-splice site of exon 6. These findings add on to the finding that local pre-mRNA secondary structures may interfere or modulate splice site recognition [13], [17], [22] and that weak splice sites need additional factors for the correct guidance of the spliceosome [11], [23], [24], [25], [26].

Taken together, our results extend the phenotypic spectrum associated with type V collagen defects and show that mutations in the type V collagen genes may cause EDS phenotypes that differ from classic EDS. Furthermore, we show that an acceptor-splice site mutation can result in different mutant transcripts, including a two-exon-skip involving an upstream exon. Since the outcome of splice site mutations is influenced by many factors, investigation of these mutations at the mRNA level is of critical importance to unravel the true splicing outcome of a specific mutation.

## Materials and Methods

### Cell culture, Steady State Collagen Labeling and Electron Microscopy

Skin biopsies were taken from the probands' inner aspect of the upper arm. Fibroblast cell culture, steady state collagen labeling and transmission electron microscopy were performed as described previously [5].

The patient and her mother participated in this study following written informed consent. This study has been approved by the Ethics Committee of the Ghent University Hospital, Ghent, Belgium.

### Molecular Analysis

Genomic DNA was extracted (DNeasy; Invitrogen) from the probands' skin fibroblasts and from a blood sample of the proband and her unaffected mother. Total RNA (TRIZOL, Invitrogen) was extracted from the probands' skin fibroblasts. For the conversion to cDNA, MMLV-RTase (Invitrogen) and random hexanucleotide primers were used [5].

*PLOD1* molecular analysis was performed at gDNA level. The presence of the common duplication of exons 10 to 16 (NG_008159.1_g.25041_33936dup) was excluded by a duplication-specific PCR as described previously [27]. Subsequently, exons and flanking intronic sequences were PCR amplified and sequenced (ABI3730XL, Applied Biosystems).

A *COL5A1* null-allele assay and mutation screening of *COL5A1* and *COL5A2* at gDNA and cDNA level respectively were performed as described previously [5]. Nucleotide numbering corresponds to the *COL5A1* reference sequence (NM_000093.3) and is according to HGMD guidelines.

To characterize the mutant mRNA transcripts, the *COL5A1* fragment encompassing exons 4 to 8 was PCR amplified (primers *COL5A1*-828F - *COL5A1*-1427R, Table 1) and cloned (TOPO-TA cloning kit, Invitrogen). Resulting clones were sequenced (ABI3730XL, Applied Biosystems).

**Table 1:** *COL5A1* N-propeptide primers.

Primer*	Sequence (5′→3′)
**cDNA cloning**	
*COL5A1*-828F	ACATCAATGGCATCATCGTG
*COL5A1*-1427R	CAGTGAACTCCCCCTCCAA
***In vitro*** ** mutagenesis**	
*COL5A1*-E7deletion-F	GAGGTCCCCGAGCCAGCTCCGCCT
*COL5A1*-E7deletion-R	AGGCGGAGCTGGCTCGGGGACCTC
*COL5A1*-E6_E7deletion-F	GACCCCAATCCAGATGAATATCCAGCTCCGCC
*COL5A1*-E6_E7deletion-R	GGCGGAGCTGGATATTCATCTGGATTGGGGTC
**Order of intron removal**	
IVS4F	TCTGGACTTTCCCCTGCTTCAAGGC
E5F	CGGGCAGCTTATGATTACTGTGAGC
IVS5F	CTGGAGGTTGCGGAAGGAAGGACAG
E6F	CGAAGACCTAGGGAAGGAGCCCAC
IVS7R	TGGCCCACTGCCCCAGACCAAAC
E8R	CGGATCGTTTCCTCAGTGAACTCC
E9R	CTTTTGGCCTTTCTCGCCCCGAG

*“IVS” denotes intervening sequence-derived oligonucleotides, “E” denotes exon-derived oligonucleotides. “F” denotes forward, “R” denotes reverse.

### Computational Algorithms and RNA Secondary Structure Prediction

The effect of the mutation on pre-mRNA splicing was evaluated using the Splice Site Prediction by Neural Network [28] and the Human Splicing Finder (HSF) [29] software. The HSF software also predicts the interaction of several splicing proteins, e.g. serine/arginine (SR) proteins, with splicing regulatory elements (SREs). The ExonScan software was used to search the *COL5A1* N-propeptide encoding region for candidate exons based on splice site strength and the presence of SREs [30]. To evaluate local pre-mRNA folding, *COL5A1* 80-mers (40 nucleotides from the end of intron 6 and 40 nucleotides from the beginning of exon 7) were folded with the RNA free energy minimization program for RNAfolding, Mfold version 3.2 [31].

### Generation of Mutant *COL5A1* N-propeptide-encoding constructs and Protein Analysis

Starting from the wild type *COL5A1* N-propeptide construct pNα1(V)-pCEP4 [12] (gift from Prof. Ruggiero, IBCP, Lyon, France), we generated the mutant constructs exon7deletion-pNα1(V)-pCEP4 and exon6_exon7deletion-pNα1(V)-pCEP4 through *in vitro* site-directed mutagenesis (QuikChange® II Lightning Site-Directed Mutagenesis kit, Stratagene Inc.) using primers listed in Table 1. The presence of the deletions was confirmed by sequencing (ABI3730XL, Applied Biosystems).

The expression and secretion of mutant α1(V)-N-propeptide proteins was evaluated by transfecting the constructs into HEK293-T cells using Lipofectamine 2000 (Invitrogen). Immunoblotting experiments were performed as described previously [7].

### Determination of Intron Removal

To determine the order of intron removal between *COL5A1* exons 5 to 9, we analyzed the splicing intermediates from nuclear pre-mRNA as reported previously. Briefly, nuclear pre-mRNA was extracted from cultured control and patient fibroblasts after incubation with actinomycin D, to stop transcription, for 0, 5, 10, 20, 40, and 60 minutes [11], [22]. PCR was performed using primers listed in Table 1. PCR fragments were analyzed by agarose gel electrophoresis or by separation on the LabChip GXII (Caliper LifeSciences) and subsequently sequenced (ABI3730XL, Applied Biosystems).

## Supporting Information

Figure S1 Pre-mRNA secondary structures of 80-mers surrounding the exon 7 acceptor-splice site. Secondary structures were calculated using Mfold. In the wild type folding, the exon 7 acceptor-splice site is easy accessible for the splicing machinery due to its presence in a loop structure. In the mutant NM_000093.3_c.925-2A>G sequence, the exon 7 acceptor-splice site is shifted towards a “stem” structure, rendering this splice site difficult to access for the spliceosome. Yellow, last 40 nucleotides of intron 6; blue, first 40 nucleotides of exon 7; green, wild type exon 7 acceptor-splice site; red, mutant exon 7 acceptor-splice site.
(TIF)
