# Supplementary figures and images for: A Novel Splice Variant in the N-propeptide of COL5A1 Causes an EDS Phenotype with Severe Kyphoscoliosis and Eye Involvement
Source: PLoS One. 2011 May 17;6(5):e20121. doi: 10.1371/journal.pone.0020121 (PMC3096658; doi:10.1371/journal.pone.0020121)

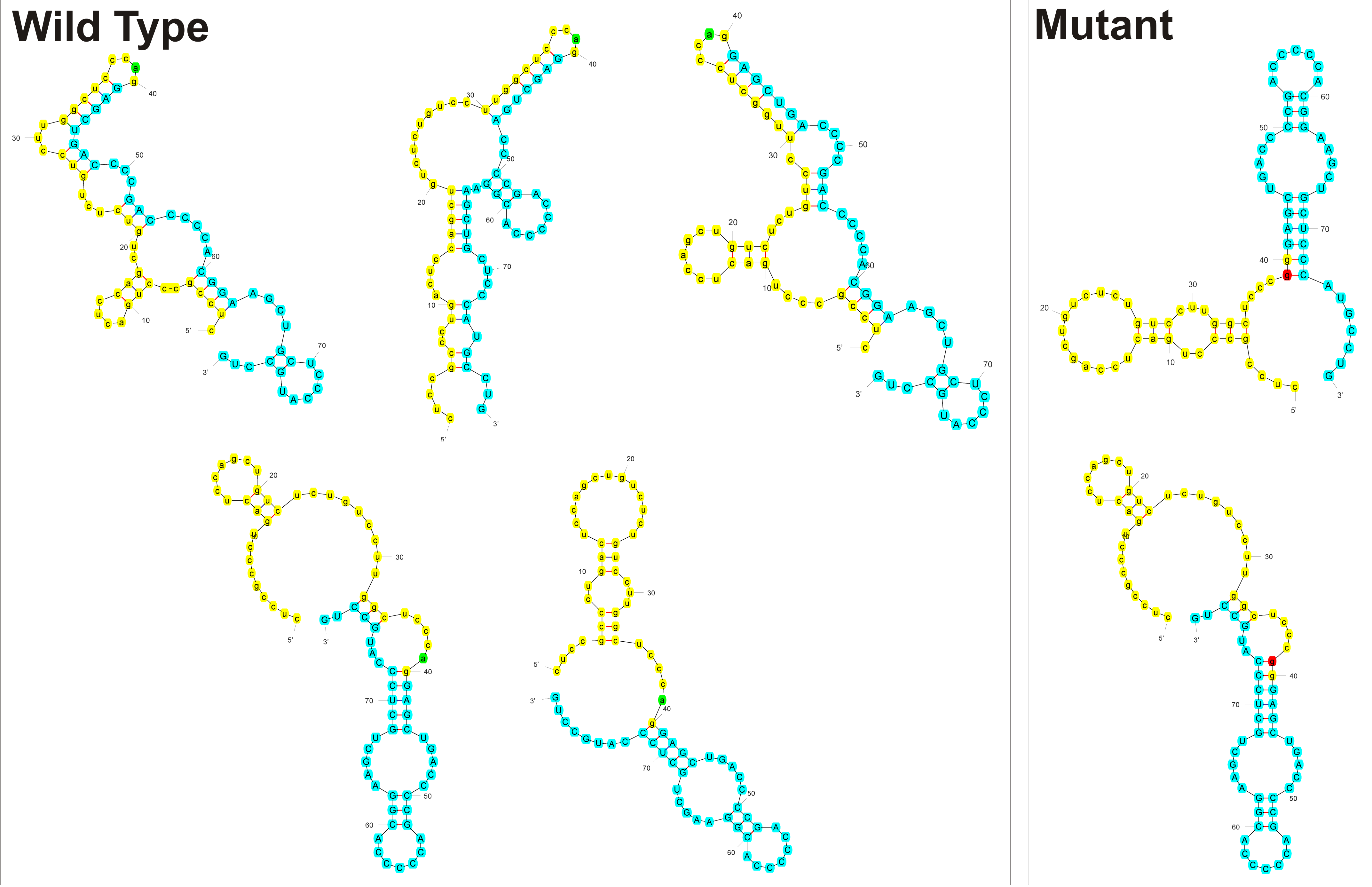

Supplement: Figure S1 — Pre-mRNA secondary structures of 80-mers surrounding the exon 7 acceptor-splice site. Secondary structures were calculated using Mfold. In the wild type folding, the exon 7 acceptor-splice site is easy accessible for the splicing machinery due to its presence in a loop structure. In the mutant NM_000093.3_c.925-2A>G sequence, the exon 7 acceptor-splice site is shifted towards a “stem” structure, rendering this splice site difficult to access for the spliceosome. Yellow, last 40 nucleotides of intron 6; blue, first 40 nucleotides of exon 7; green, wild type exon 7 acceptor-splice site; red, mutant exon 7 acceptor-splice site. (TIF) [file pone.0020121.s001.tif]
